# Improving the Accuracy of Multi-Breed Prediction in Admixed Populations by Accounting for the Breed Origin of Haplotype Segments

**DOI:** 10.3389/fgene.2022.840815

**Published:** 2022-03-24

**Authors:** Markus Schmid, Joana Stock, Jörn Bennewitz, Robin Wellmann

**Affiliations:** Institute of Animal Science, Department of Animal Genetics and Breeding, University of Hohenheim, Stuttgart, Germany

**Keywords:** admixed population, multi-breed genomic prediction, BOA model, cattle, allele origin

## Abstract

Numerically small breeds have often been upgraded with mainstream breeds. This historic introgression predisposes the breeds for joint genomic evaluations with mainstream breeds. The linkage disequilibrium structure differs between breeds. The marker effects of a haplotype segment may, therefore, depend on the breed from which the haplotype segment originates. An appropriate method for genomic evaluation would account for this dependency. This study proposes a method for the computation of genomic breeding values for small admixed breeds that incorporate phenotypic and genomic information from large introgressed breeds by considering the breed origin of alleles (BOA) in the evaluation. The proposed BOA model classifies haplotype segments according to their origins and assumes different but correlated SNP effects for the different origins. The BOA model was compared in a simulation study to conventional within-breed genomic best linear unbiased prediction (GBLUP) and conventional multi-breed GBLUP models. The BOA model outperformed within-breed GBLUP as well as multi-breed GBLUP in most cases.

## Introduction

The efficiency of breeding programs for local breeds is often compromised by the limited number of individuals and has resulted in a decreasing competitiveness with high yielding breeds, especially with the advent of genomic selection (GS). In GS, large reference populations are required to accurately predict breeding values of the individuals (Goddard and Hayes 2009) and are therefore difficult to establish in small local breeds. In order to improve the performance of local breeds, sires of closely related high-yielding breeds were frequently used in the past and genetic gain has been generated by introgression. Such strategies increase the genetic relatedness between breeds because a certain number of alleles of the high yielding breed segregate within the target breed after introgression.

Several studies were conducted using different approaches to enable GS in numerically small breeds using the reference population of a second breed (across-breed prediction) or extending the own reference population by adding the reference population of the second breed (multi-breed prediction) as reviewed by Lund et al. (2014, 2016). The major findings were that across-breed prediction is often not suitable to improve the accuracy of prediction and that the benefit of multi-breed reference populations strongly depends on the relatedness between the breeds and density of the SNP panels. A substantial increase in accuracy can only be expected when the breeds are closely related and the number of SNPs is high to capture across-breed linkage disequilibrium (LD) between markers and QTLs. However, variation of LD as well as differences of allelic effects across populations limit the application of such approaches. Different models were proposed accounting for breed-specific effects (e.g., Makgahlela et al., 2012; Thomasen et al., 2013; Hamidi and Rekaya., 2015; van den Berg et al., 2020) and differences in LD (Rahimi et al., 2020) in the field of multi-breed dairy cattle evaluation. One way is to assign the breed origin of alleles (BOA) (Wellmann 2019; Vandenplas et al., 2016) that allows for models assuming SNP effects to be different but correlated across breeds. Such models were applied to simulated and real datasets of crossbred or admixed populations in cattle (Karaman et al., 2021) as well as other livestock (e.g., Duenk et al., 2019) or plant species (Rio et al., 2020) and are reviewed in Stock et al. (2020) and Duenk et al. (2021). The studies have shown that considering BOA has the potential to increase the accuracy of multi-breed GS.

In many numerical small dairy cattle breeds sires from a large and high yielding breed were used in order to speed up genetic gain in the small breed. This resulted in some cases in a substantial amount of introgressed genes and in a mosaic-like haplotype pattern with a mix of native and introgressed haplotypes. For example, in the German Angler breed located in the northern part of Germany, admixture plays a substantial role in the population structure and the proportion of migrant alleles from other breeds is remarkable (Addo et al., 2019, Wang et al., 2017a,b, Schmidtmann et al., 2021). A very close relationship to the Holstein Friesian breed, especially the Red Holstein breed, was observed (Wang et al., 2017b). A similar level of admixture was observed for the German Vorderwald breed, where the genetic progress was mostly driven by the introgressed genes (Hartwig et al., 2014; Hartwig et al., 2015). For these kinds of breeds, a genomic model that considers the mosaic pattern of the haplotype structure would be beneficial in multi-breed genomic evaluations.

This study proposes a method for the computation of genomic breeding values for small admixed breeds that incorporates phenotypic and genomic information from large introgressed breeds. The start and the end of the introgression events are considered to be in the past, which is applicable to many small local admixed breeds. A multi-breed BOA model is derived for multi-breed genomic selection that is suitable for application when the individuals have fragmented genomes. It classifies haplotype segments according to their origins and assumes different SNP effects for the different origins.

For validation, it was compared with models that did not consider the breed-origin of QTL alleles. All models were applied to simulated datasets. In the simulation, the genotypes of the small admixed breed were derived from German Angler cattle, while the genotypes of the introgressed breed were derived from German Holstein cattle. Different scenarios were investigated in which the number of genotyped animals of the target breed, i.e., the numerically small Angler breed, varied, while the number of genotyped animals of the large introgressed breed, i.e., the German Holstein, remained constant.

## Materials and Methods

### Simulation

The data basis for the simulation study were 50k SNP-chip (Illumina BovineSNP50 BeadChip, Illumina Inc., San Diego, CA) genotypes of Angler (AN) (Wang et al., 2017a) and Holstein (HF) (Streit et al., 2013) individuals from the German population. Starting with the base generation, one further generation was simulated for each breed according to the simulation protocol of Stock et al. (2021) with R-package x-breed (Esfandyari and Sørensen 2017). The resulting simulated HF dataset (simHF) consisted of 6,000 individuals and the simulated Angler data set contained 3,000 individuals.

Several subsets of the total Angler data set were sampled to mimic different population sizes for the small breed. Subset simAN1 consisted of 750 individuals, simAN2 consisted of 1,500 individuals and simAN3 contained all 3,000 simulated Angler individuals. The different simAN populations are referred to as breed size scenarios. The sample sizes represent 12.5, 25 and 50% of the number of simHF individuals. In each of the subsets, all sires had the same number of offspring.

From the 23,448 SNPs that segregated in both breeds, 1,000 SNPs were randomly selected as QTLs, while the remaining SNPs were used as markers for genomic prediction.

The QTL effects for the simAN datasets and the simHF dataset were correlated. The additive effects $a_{q}^{\text{HF}},a_{q}^{\text{AN}}$ of QTL q were correlated and normally distributed with

$$\left(\begin{array}{c} a_{q}^{\text{HF}}\\ a_{q}^{\text{AN}} \end{array}\right)∼N_{2}\left(\left(\begin{array}{c} 0\\ 0 \end{array}\right),\sigma_{A}^{2}\left(\begin{array}{cc} 1 & 0.95\\ 0.95 & 1 \end{array}\right)\right)$$

Hence, the correlation of QTL effects between the two simulated breeds was 0.95. Dominance was not modelled. The additive effects were scaled to represent a trait with an additive variance of $V_{A}\;\approx \;0.3$ in each of the pure breeds. The additive genetic variance was calculated using standard formulas (Falconer and Mackay 1996). The phenotypes were obtained by adding normally distributed errors to the true genotypic values. The error variance was chosen to obtain a phenotypic variance of $V_{P}=1$ . The entire simulation was independently repeated ten times.

### True Breeding Values

The true breeding values (TBV) were calculated as

$$TGV_{ik}=\underset{q\in Q}{\sum }\underset{k\in \left\{\text{AN},\text{HF}\right\}}{\sum }Z_{Aiq}^{k}a_{q}^{k}$$

where $a_{q}^{k}$ is the additive effect of QTL q when the haplotype segment containing the QTL originated from breed k∈{AN, HF} . The allele content $Z_{Aiq}^{k}\in \left\{0,1,2\right\}$ equals the number of copies of the alternative allele in individual i that originate from breed k . The R-package optiSel (Wellmann 2019) was used to assign the QTL alleles from the simAN data set to either the Angler or the Holstein breed in a segment-based approach. Considered segments consisted of minimum 20 consecutive markers and had a length of ≥1.5 Mb. No Angler cattle were introgressed into Holstein cattle, so the summand corresponding to k=AN equals zero in the simHF data set.

### Genomic Prediction

The BOA model was compared with two conventional methods for the prediction of genomic breeding values, which are within-breed prediction with GBLUP for the simulated Angler cattle, and a multi-breed prediction with GBLUP.

#### The Breed Origin of Alleles model

It is assumed that genotypes and phenotypes from several breeds or crosses are available, which includes the target breed. The number of SNP is denoted as M , and the total number of individuals as N . The genotypes are phased, so each individual i has a maternal haplotype $h_{i}^{♀}$ and a paternal haplotype $h_{i}^{♂}$ . The binary coded alleles of individual i at SNP m are denoted as $h_{im}^{♀},\;h_{im}^{♂}\in \left\{0,\;1\right\}$ . The origins $o_{im}^{♂},\;o_{im}^{♀}\;\in \left\{1,\;...,\text{ K}\right\}$ of all positions m in all haplotypes $h_{i}^{♀},$ $h_{i}^{♂}$ are determined, whereby K denotes the number of possible origins. Each origin can be considered as a genetic group, whereby the first genetic group is the target breed. The indicators for genetic group k are denoted as

$$\delta_{kim}^{♂}=\;\{\begin{array}{c} 1\;\;if\;o_{im}^{♂}=k\\ 0\;otherwise \end{array}$$

and

$$\delta_{kim}^{♀}=\;\{\begin{array}{c} 1\;if\;o_{im}^{♀}=k\\ 0\;otherwise \end{array}.$$

The model equation for the phenotypic value of individual i is

$$y_{i}=\;\overset{K}{\underset{k=1}{\sum }}c_{ik}\beta_{k}+\;\overset{K}{\underset{k=1}{\sum }}\overset{M}{\underset{m=1}{\sum }}\left(h_{im}^{♂}\delta_{kim}^{♂}+\;h_{im}^{♀}\delta_{kim}^{♀}\right)a_{m}^{k}+\;e_{i}$$

where $c_{ik}\in \left[0,\;1\right]$ is the genetic contribution, individual i has from genetic group k , $\beta_{k}$ is the fixed effect of genetic group k , $a_{m}^{k}$ is the normally distributed additive effect of marker m in genetic group k , and $e_{i}$ is the residual. The model equation in matrix form is

$$y=X\beta +\sum_{k=1}^{K}Z_{A}^{k}a_{k}+e$$

where $\beta ={\left(\beta_{1},...,\beta_{K}\right)}^{T}\;$ is the vector of fixed effects with N × K design matrix X , where $X_{ik}=c_{ik}$ . The M -vector $a_{k}$ of random SNP effects for genetic group k has design matrix $Z_{A}^{k}$ , where $Z_{Aim}^{k}=h_{im}^{♂}\;\delta_{kim}^{♂}+h_{im}^{♀}\delta_{kim}^{♀}$ is the number of copies of the alternative allele that originate from genetic group k . The vector e with residuals has covariance matrix $R\;=\;\sigma_{e}^{2}I.\;$ The covariance matrix of the KM -vector $a={\left(a_{1}^{T},...,a_{K}^{T}\right)}^{T}$ is

$$D=Cov\left(a\right)=\left(\begin{array}{ccc} \sigma_{A1}^{2}I & \cdots & \sigma_{AK1}I\\ \vdots & \ddots & \vdots \\ \sigma_{AK1}I & \cdots & \sigma_{AK}^{2}I \end{array}\right)=\Sigma ⊗I$$

with

$$\sum =\left(\begin{array}{ccc} \sigma_{A1}^{2} & \cdots & \sigma_{AK1}\\ \vdots & \ddots & \vdots \\ \sigma_{AK1} & \cdots & \sigma_{AK}^{2} \end{array}\right)\;$$

Alternative representations of the model and the mixed model equations are given in the Supplementary Appendix.

The BOA model requires the breed origins $o_{im}^{♂}$ and $o_{im}^{♀}$ of the haplotypes as input parameters. The R-package optiSel (Wellmann 2019) was used to assign all marker haplotype segments from the simAN data set to either the Angler or the Holstein breed. Considered segments consisted of minimum 20 consecutive markers and had a length of ≥1.5 Mb.

The covariance matrix Σ of the marker effects of the two pure breeds was needed as an input parameter. The correlation was determined by a grid search for each replicate and breed size. A 5-fold cross-validation was conducted for each candidate value, and the correlation was chosen for further analyses that maximized the accuracy of the predictions with the BOA model. As the resulting correlation values were similar across all replicates and breed size scenarios and the mean value was approximately 0.75, this value ( r=0.75 ) was chosen as input parameter for all subsequent evaluations. It was expected that such an approach would avoid an overfitting of the model. The correlation matrix for the marker effects was multiplied by the estimated variance of the allele substitution effects to obtain the covariance matrix of the marker effects.

#### Alternative models

For within-breed genomic prediction we used the model

$$y=\;\beta 1+\;Z_{A}a+\;e,$$

where the M -vector a of random SNP effects has covariance matrix $D=\sigma_{a}^{2}I$ and design matrix $Z_{A}$ , where $Z_{Aim}$ is the number of copies of the alternative allele at marker m in individual i . The vector e with residuals has covariance matrix $R\;=\;\sigma_{e}^{2}I.\;$ For multi-breed genomic prediction, the same model was used. We did not include a breed effect because no breed effect was simulated.

The SNP markers for genomic prediction were chosen as follows. From the 22,448 SNPs that were not chosen as QTLs, all SNPs that segregated with a minor allele frequency (MAF) <0.03 within one of the simulated breeds and SNPs that did not segregate in both breeds were omitted. Across all replicates, on average 21,670 SNPs remained and were used for genomic prediction.

### Cross Validation

The genomic predictions were done separately for each breed-size scenario and each replicate. The accuracies of prediction were assessed by a 5-fold cross validation. The individuals of the respective simAN dataset were assigned to five different classes such that individuals from different classes had no sires in common. Hence, each class included the offspring of 10 sires. In each cross-validation cycle, one class was used as the validation set, and the four remaining classes were used as the reference population.

For multi-breed GBLUP and for the BOA model, the respective simAN reference set was joined with the simHF individuals. Consequently, the number of individuals from the simAN population in the reference population varied, while the number of simHF individuals was constant.

An overview on the sample sizes is given in Table 1. The reference populations for within-breed prediction consisted of 600, 1,200, and 2,400 simAN individuals, respectively. The reference populations for multi-breed prediction were enlarged by the 6,000 simHF individuals. The proportions of simAN individuals in the multi-breed reference population were thus 9, 17 and 29% for the simAN1, simAN2 and simAN3 scenario, respectively.

**TABLE 1:** Numbers of genotyped animals in reference and validation sets for the three investigated scenarios.

Scenario	*n*	Validation set	Reference set 1 (within-breed)	Reference set 2 (multi-breed)	simAN % (multi-breed)
simAN1	750	150	600	6,600	9.09
simAN2	1,500	300	1,200	7,200	16.67
simAN3	3,000	600	2,400	8,400	28.57

The size of the simulated Angler data set (n), the number of simulated Angler individuals in each validation set (Validation Set), the number of simulated Angler individuals in each reference set for within-breed prediction (Reference Set 1), the number of simulated Angler and simulated Holstein Friesian individuals in the reference set for multi-breed prediction (Reference Set 2), and the proportion of simulated Angler individuals in the multi-breed reference set (simAN %).

The marker effects were estimated with all three models for each cross-validation cycle.

### Estimated Breeding Values

The genomic breeding values of the individuals in the validation set were computed for the BOA model as

$$GEBV_{i}=\underset{m\in M}{\sum }\sum_{k\in \left\{\text{AN},\text{HF}\right\}}Z_{Aim}^{k}{\hat{a}}_{m}^{k},$$

where M is the set of SNP markers, ${\hat{a}}_{m}^{k}$ is the estimated SNP effect of marker m that is used for haplotype segments originating from genetic group k , and the allele content $Z_{Aim}^{k}\in \left\{0,1,2\right\}$ equals the number of copies of the alternative allele in individual i that originate from genetic group k . For the other models, the genomic breeding values were estimated as

$$GEBV_{i}=\sum_{m\in M}Z_{Aim}{\hat{a}}_{m},$$

where ${\hat{a}}_{m}$ is the estimated SNP effect of marker m , and the allele content $Z_{Aim}\in \left\{0,1,2\right\}$ equals the number of copies of the alternative allele in individual i at marker m .

### Prediction Accuracies

For each method, the prediction accuracy was calculated as the correlation between the GEBVs and the TBVs of the validation individuals. The accuracies presented in the results are the averages, taken over all cross-validation cycles and replicates.

## Results

The mean proportion of SNPs with Holstein origin across all replicates was 0.157 ± 0.007. Table 2 shows the results of the model comparison for all investigated breed-size scenarios. In general, the prediction accuracies increased with increasing size of the reference population. The BOA model provided the highest accuracies for simAN1 and simAN2, whereas it showed the same mean accuracy as within-breed GBLUP for simAN3. Multi-breed GBLUP was inferior to the other models in simAN1 and simAN3. Within-breed GBLUP resulted in the lowest accuracies in the medium-sized reference population scenario simAN2. The standard deviations (SD) of the accuracies were highest using the small reference set, while it showed the smallest SD values in the medium-sized reference set. The standard errors of the accuracies were relatively small (0.011–0.017).

**TABLE 2:** Mean accuracies of genomic prediction in the simulated breed size scenarios.

Model	Reference set	simAN1			simAN2			simAN3		
		Mean	sd	se	Mean	sd	Se	Mean	sd	se
BOA	simAN + simHF	0.407	0.110	0.016	0.492	0.074	0.011	0.546	0.085	0.012
GBLUP	simAN + simHF	0.387	0.118	0.017	0.486	0.078	0.011	0.541	0.092	0.013
GBLUP	simAN	0.401	0.114	0.016	0.477	0.080	0.011	0.546	0.083	0.012

The mean, standard deviation (sd) and standard error (se) of the 5-fold cross-validation across the 10 simulated replicates are given for the breed size scenarios simAN1, simAN2 and simAN3 and for the BOA and the GBLUP models. Column 2 indicates whether a multi-breed reference set or a within-breed reference set was used.

## Discussion

It was shown in this study that a multi-breed genomic prediction with the proposed BOA model can increase the accuracies of the GEBVs for numerically small admixed populations over multi-breed and within-breed genomic predictions with GBLUP under certain conditions. The model considers the breed origin of each haplotype in individuals with admixed genomes consisting of native and introgressed haplotype segments. This makes it especially interesting for numerical small breeds with historic introgression from high yielding breeds, as it was observed e.g., in the German Angler or Vorderwald breed (Addo et al., 2019; Wang et al., 2017a; Wang et al., 2017b; Schmidtmann et al., 2021; Hartwig et al., 2014, 2015).

The simulated QTL positions were the same in both breeds. Recent mutations that could have created new QTLs were ignored. The QTL effects of both genetic groups were assumed to be highly correlated with a correlation of 0.95. The QTL positions were chosen from the 50k chip, so the simulated QTLs are common variants whereas a large fraction of the additive variance is expected to come from rare QTL variants (e.g., Kemper and Goddard 2012; Visscher et al., 2017). This can compromise a direct transition of the simulation results to real data.

The LD structures in the simulation are expected to be similar to those investigated in real populations (Qanbari et al., 2010; Addo et al., 2019; Schmidtmann et al., 2021) because only one generation was simulated, so recombination could occur only within one meiotic division. Although the QTL effects were highly correlated, the correlation of the marker effects was only 0.75. The reason for the relatively low correlation of marker effects is that the QTLs were excluded from the marker set. Therefore, the effect of a single QTL is captured by several markers and the LD between markers and QTLs is different in both genetic groups. The shorter ranges of LD in admixed populations like the German Angler compared with other breeds (Addo et al., 2019; Schmidtmann et al., 2021) contributes to the observed low correlation. A higher correlation between marker effects might be observed when more dense marker panels would be used and a heavy-tailed distribution of marker effects would be assumed.

This paper focused on methods to improve GS in small admixed populations. It compared the prediction accuracies of various methods in a simulated population that had a similar LD structure as the target breeds. A detailed quantification of the impact of influencing factors (e.g., LD and its consistency across populations, or the relatedness and genetic correlation between the populations) on the accuracies was beyond the scope of the study. The study explicitly focused on admixed populations where the events of introgression were in the past and breeding programs aim at reducing migrant contributions from other (high yielding) breeds or at least keep the amount of migrant contributions constant (e.g., Wang et al., 2017b). Therefore, the impact of the approach on crossbred individuals was not determined. But still, models that include BOA information have been shown to be beneficial for crosses (e.g., Duenk et al., 2019).

In this study, the multi-breed BOA model was compared with conventional multi-breed GBLUP and within-breed GBLUP. The multi-breed BOA approach led to an increase in the accuracy of the genomic breeding values when the number of genotyped AN individuals was small and medium, and showed similar results as the within-breed GBLUP method for the large reference sets. The difference between the prediction accuracies of the models, however, tend to decrease with an increasing number of genotyped AN individuals. The multi-breed prediction with GBLUP was not superior to within-breed prediction when the number of genotyped AN individuals was large. The reason is possibly that the multi-breed GBLUP model assumes a perfect correlation between the marker effects of both breeds. This assumption was certainly violated in the simulation. The BOA model, which accounts for the correlation between marker effects, could improve upon single-breed evaluations and outperformed multi-breed GBLUP in all cases.

For the prediction of genomic breeding values of Angler in practice, to date, a joint reference population of several Scandinavian red dairy breeds (i.e., Danish Red, Norwegian Red, Swedish Red, and Finnish Ayrshire) is used. To increase the accuracies of the GEBVs for Angler, about 170 genotyped and progeny-tested German Angler bulls have been included to this reference set as well (private communication RSHeG, 2021). Hence, the findings of the study in scenario simAN1 might be most relevant for the current Angler cattle breeding program.

In the past decades, the Angler breed has been upgraded with other breeds, such as Red Holstein and Holstein Friesian to increase its economic value. This has led to relatively high kinships between them (Wang et al., 2017b). However, in this study only Holstein Friesian genotypes were available and considered, and thus the total amount of introgression was probably not detected completely. In addition, the available Holstein Friesian genotypes originated from the current population, which might have also biased the categorization of the native parts of the genome. The use of the most closely related introgressed breed is expected to bear the greatest potential in multi-breed predictions in the target breed when applying BOA models and should therefore preferably be used if applicable. This was not shown here as such datasets were not available. At an animal level, the proposed BOA model considers the genetic connectedness of individuals from both breeds. The closer individuals of the high yielding breed are related with the individuals of the target breed, the more informative they are for multi-breed prediction and thus contribute more to the accuracy of breeding value estimation. Generally, multi-breed prediction is increasingly beneficial when applied to high density marker information or whole-genome sequence data (Lund et al., 2014), however, such datasets are mostly not available in cost-efficient breeding programs of small local cattle populations.

## Conclusion

A multi-breed genomic prediction with the proposed BOA model increased the accuracies of the estimated genomic breeding values for numerically small admixed populations over multi-breed and within-breed genomic predictions with GBLUP. The BOA model assumes that the additive effect of an allele depends on the genetic group from which the respective haplotype segment originates. It is of special interest for multi-breed genomic predictions for numerically small breeds with past introgression from high yielding breeds.

## Data Availability

The original contributions presented in the study are included in the article/Supplementary Material, further inquiries can be directed to the corresponding author.
