# Supplementary material for: Improving the Accuracy of Multi-Breed Prediction in Admixed Populations by Accounting for the Breed Origin of Haplotype Segments
Source: Front Genet. 2022 Mar 24;13:840815. doi: 10.3389/fgene.2022.840815 (PMC8987492; doi:10.3389/fgene.2022.840815)
Supplement: Supplementary file 1 [file DataSheet1.docx]

**Improving the accuracy of multi-breed prediction in admixed populations by accounting for the breed origin of haplotype segments**

Markus Schmid^1^*, Joana Stock^1^, Jörn Bennewitz^1^, Robin Wellmann^1^

^1^ Institute of Animal Science, Department of Animal Genetics and Breeding, University of Hohenheim, Garbenstrasse 17, 70599 Stuttgart, Germany.

* Correspondence:

Markus Schmid

Markus_schmid@uni-hohenheim.de

# Appendix

The BOA model has the alternative animal-model representation

$y = X\beta+ \sum_{k=1}^{K} u_{k}+e$,

where $u_{k}= Z_{A}^{k}a_{k}$ is the $N$-vector with animal effects from genetic group $k$. The $KN$-vector $u{= ({u_{1}}^{T} , ...,{u_{K}}^{T})}^{T}$ with all animal effects has the representation

$$u = \left( \begin{matrix} Z_{A}^{1}a_{1} \\ \vdots\\ Z_{A}^{k}a_{k} \end{matrix} \right)= \tilde{Z}_{A}a$$

with $KN \times KM$-matrix

$\tilde{Z}_{A}= \left( \begin{matrix} Z_{A}^{1} & 0 & \cdots& 0 \\ 0 & Z_{A}^{2} & \ddots& \vdots\\ \vdots& \ddots& \ddots& 0 \\ 0 & \cdots& 0 & Z_{A}^{K} \end{matrix} \right)$.

The covariance matrix of $u$ is

$$G=Cov\left( u \right) = Cov\left( \tilde{Z}_{A}a \right) = \tilde{Z}_{A} Cov\left( a \right) \tilde{Z}_{A}^{T} = \tilde{Z}_{A} \left( \Sigma\otimes I \right) \tilde{Z}_{A}^{T} = \left( \begin{matrix} Z_{A}^{1} & \cdots& 0 \\ \vdots& & \vdots\\ 0 & \cdots& Z_{A}^{K} \end{matrix} \right)\left( \begin{matrix} \sigma_{A1}^{2}I & \cdots& \sigma_{A1K}I \\ \vdots& & \vdots\\ \sigma_{AK1}I & \cdots& \sigma_{AK}^{2}I \end{matrix} \right)\left( \begin{matrix} Z_{A}^{1T} & \cdots& 0 \\ \vdots& & \vdots\\ 0 & \cdots& Z_{A}^{KT} \end{matrix} \right)$$

$$= \left( \begin{matrix} \sigma_{A1}^{2}Z_{A}^{1}Z_{A}^{1T} & \cdots& \sigma_{A1K}Z_{A}^{1}Z_{A}^{KT} \\ \vdots& & \vdots\\ \sigma_{A1K}Z_{A}^{K}Z_{A}^{1T} & \cdots& \sigma_{AK}^{2}Z_{A}^{K}Z_{A}^{1T} \end{matrix} \right)$$

Thus, the mixed linear model has the alternative representation

$$y = X\beta+ Zu + e$$

with $N \times KN$-matrix $Z = (I, ..., I)$. Henderson’s mixed model equations are

$\left( \begin{matrix} X^{T}R^{-1}X & X^{T}R^{-1}Z \\ Z^{T}R^{-1}X & Z^{T}R^{-1}Z+G^{-1} \end{matrix} \right)\left( \hat{\begin{aligned} \beta\\ \hat{u} \end{aligned}} \right)=\left( \begin{aligned} X^{T}R^{-1}y \\ Z^{T}R^{-1}y \end{aligned} \right)$,

where $R=\sigma_{e}^{2}I$. They can be simplified as

$\left( \begin{matrix} X^{T}X & X^{T}Z \\ Z^{T}X & Z^{T}Z+\sigma_{e}^{2}G^{-1} \end{matrix} \right)\left( \hat{\begin{aligned} \beta\\ \hat{u} \end{aligned}} \right)=\left( \begin{aligned} X^{T}y \\ Z^{T}y \end{aligned} \right)$.

The alternative model representation as an SNP-model is

$y = X\beta+ Z_{A}a+ e$,

where $Z_{A} = (Z_{A}^{1}, ..., Z_{A}^{K})$, which provides the following alternative mixed model equation:

$\left( \begin{matrix} X^{T}X & X^{T}Z_{A} \\ Z_{A}^{T}X & Z_{A}^{T}Z_{A}+\sigma_{e}^{2}D^{-1} \end{matrix} \right)\left( \hat{\begin{aligned} \beta\\ \hat{a} \end{aligned}} \right)=\left( \begin{aligned} X^{T}y \\ Z_{A}^{T}y \end{aligned} \right)$,

where $D^{-1} = \Sigma^{-1} \otimes I$. The expanded version of this equation is

$$\left( \begin{matrix} X^{T}X & X^{T}Z_{A}^{1} & \cdots& X^{T}Z_{A}^{K} \\ Z_{A}^{1T}X & Z_{A}^{1T}Z_{A}^{1}+\sigma_{e}^{2}\Sigma_{11}^{-1}I & \cdots& Z_{A}^{1T}Z_{A}^{K}+\sigma_{e}^{2}\Sigma_{1K}^{-1}I \\ \vdots& \vdots& \cdots& \vdots\\ Z_{A}^{KT}X & Z_{A}^{KT}Z_{A}^{1}+\sigma_{e}^{2}\Sigma_{K1}^{-1}I & \cdots& Z_{A}^{KT}Z_{A}^{K}+\sigma_{e}^{2}\Sigma_{KK}^{-1}I \end{matrix} \right)\left( \hat{\begin{aligned} \beta\\ \hat{a}_{1} \\ \vdots\\ \hat{a}_{K} \end{aligned}} \right)=\left( \begin{aligned} X^{T}y \\ Z_{A}^{1T}y \\ \vdots\\ Z_{A}^{KT}y \end{aligned} \right)$$
